# Effect of sodium–glucose cotransporter-2 inhibitors on aldosterone-to-renin ratio in diabetic patients with hypertension: a retrospective observational study

**DOI:** 10.1186/s12902-020-00656-8

**Published:** 2020-11-30

**Authors:** Toshitaka Sawamura, Shigehiro Karashima, Satoshi Nagase, Hidetaka Nambo, Eiko Shimizu, Takuya Higashitani, Daisuke Aono, Azusa Ohbatake, Mitsuhiro Kometani, Masashi Demura, Kenji Furukawa, Yoshiyu Takeda, Takashi Yoneda

**Affiliations:** 1 Division of Endocrine and Diabetes, Department of Internal Medicine, Kanazawa University Graduate School of Medicine, 13-1 Takaramachi, Kanazawa, Ishikawa 920-8641 Japan; 2 Division Department of Diabetes and Endocrinology and Internal Medicine, Fukui Prefectural Hospital, 2-8-1 Yotsui, Fukui, Fukui 910-8526 Japan; 3 Department of Laboratory Sciences, Faculty of Health Sciences, Kanazawa University, Kanazawa, Japan; 4 School of Electrical, Information and Communication Engineering, College of Science and Engineering, Kanazawa University, Kanazawa, Japan; 5 Department of Hygiene, Kanazawa University Graduate School of Medicine, 13-1 Takaramachi, Kanazawa, Ishikawa 920-8641 Japan; 6 Health Care Center, Japan Advanced Institute of Science and Technology, 1-1 Asahidai, Nomi, Ishikawa 923-1292 Japan; 7 Division Department of Internal Medicine, Houju memorial hospital, 11-71 Midorigaoka, Nomi, Ishikawa 923-1226 Japan; 8 Institute of Liberal Arts and Science, Kanazawa University, 13-1 Takara-machi, Kanazawa, Ishikawa 920-8641 Japan; 9 Department of Health Promotion and Medicine of the Future, Kanazawa University, 13-1 Takaramachi, Kanazawa, Ishikawa 920-8641 Japan

**Keywords:** SGLT2 inhibitor, Aldosterone-to-renin ratio, Renin-angiotensin-aldosterone system, Primary aldosteronism, Diabetes, Hypertension

## Abstract

**Background:**

Plasma aldosterone-to-renin ratio (ARR) is popularly used for screening primary aldosteronism (PA). Some medications, including diuretics, are known to have an effect on ARR and cause false-negative and false-positive results in PA screening. Currently, there are no studies on the effects of sodium–glucose cotransporter-2 (SGLT2) inhibitors, which are known to have diuretic effects, on ARR. We aimed to investigate the effects of SGLT2 inhibitors on ARR.

**Methods:**

We employed a retrospective design; the study was conducted from April 2016 to December 2018 and carried out in three hospitals. Forty patients with diabetes and hypertension were administered SGLT2 inhibitors. ARR was evaluated before 2 to 6 months after the administration of SGLT2 inhibitors to determine their effects on ARR.

**Results:**

No significant changes in the levels of ARR (90.9 ± 51.6 vs. 81.4 ± 62.9) were found. Body mass index, diastolic blood pressure, heart rate, fasting plasma glucose, and hemoglobin A1c were significantly decreased by SGLT2 inhibitors. Serum creatinine was significantly increased.

**Conclusion:**

SGLT2 inhibitor administration yielded minimal effects on ARR and did not increase false-negative results in PA screening in patients with diabetes and hypertension more than 2 months after administration.

**Supplementary Information:**

The online version contains supplementary material available at 10.1186/s12902-020-00656-8.

## Background

Primary aldosteronism (PA) is the most common form of secondary hypertension. It is characterized by the inappropriate production of aldosterone and accounts for 5–10% of all patients with hypertension [1]. Patients with PA experience more cardiovascular events and cardiovascular mortality than those with essential hypertension (EHT) [2]. PA is commonly associated with impaired glucose tolerance (IGT), which occurs in almost 50% of individuals with PA [3, 4]. In addition, the prevalence of PA is reported to be 11.3–14% in patients with diabetes and hypertension [5, 6], which is greater than that found in all patients with hypertension. Treatment of PA using surgery or aldosterone antagonists prevents the progression of cardiovascular and renal complications [7–9] and improves insulin resistance in patients with IGT and diabetes [10].

Aldosterone-to-renin ratio (ARR) is commonly used to screen for PA. The national guidelines of the Japan Endocrine Society and the Endocrine Society recommend screening for PA using ARR [11, 12]. However, several anti-hypertensive agents affect the secretion of aldosterone and renin, which may complicate the interpretation of ARR and cause either false-positive or false-negative results [13–15]. For example, diuretics could affect renin and aldosterone production by reducing renal blood flow, leading to a decrease in ARR.

Sodium–glucose cotransporter-2 (SGLT2) inhibitors are a new class of diabetes medications that have diuretic effects. SGLT links one glucose to one sodium ion for transportation into the proximal tubule cell of the kidney. SGLT2 inhibitors provide an insulin-independent reduction in hemoglobin A1c (HbA1c) levels, with potential additional benefits, such as body weight loss, natriuresis, and osmotic diuresis [16, 17]. In type 2 diabetic patients, increased urinary sodium excretion has been observed during the early phase of treatment with canagliflozin [18–20] and empagliflozin [21]. Diuresis induced by SGLT2 inhibitors may activate the renin-angiotensin-aldosterone system (RAAS). The elucidation of whether the diuretic effect of SGLT2 inhibitors can change ARR values for PA screening in patients with diabetes and hypertension serves as a significant clinical issue. It is recommended that ARR be measured without anti-hypertensive agents or with an alpha-blocker and Ca-blocker, as they have little effects on the ARR in PA screening [11, 12]. We examined the effects of SGLT2 inhibitors on ARR in patients with diabetes and hypertension.

## Methods

We adopted a retrospective design, and the study was conducted in patients with diabetes and hypertension in either of the following three hospitals: Kanazawa University Hospital, Fukui Prefectural Hospital, and Houju Memorial Hospital from April 2016 to December 2018. The study was approved by the ethics committees of the Kanazawa University Hospital (No. 2015185), Fukui Prefectural Hospital (No. 17–85), and Houju Memorial Hospital (No. 16–19) with a waiver of consent being obtained from all the ethics committees. All procedures were performed in accordance with the 1964 Helsinki Declaration and its later amendments. The inclusion criteria were as follows: (1) patients with type 2 diabetes and hypertension aged more than 20 years and who was administrated SGLT2 inhibitor, (2) plasma renin activity (PRA) and plasma aldosterone concentration (PAC) was evaluated before (within 3 months before SGLT2 administration) and after (2 to 6 months after SGLT2 administration) administration of SGLT2 inhibitor, and (3) patients whose anti-hypertensive and anti-diabetic agents were not changed between the period of 3 months before the first evaluation and second evaluation points of PRA and PAC. The exclusion criteria were as follows: (1) secondary hypertension including PA, and (2) patients taking mineralocorticoid receptor antagonists (MRA), such as spironolactone and eplerenone. Data were collected routinely within the setting of clinical practice according to standard procedures. Informed consent was obtained in the form of opt-out on the web site. The datasets during the current study are available from the corresponding author on reasonable request.

### Data collection at baseline and the intervention

Systemic blood pressure (SBP), diastolic blood pressure (DBP), heart rate (HR), body weight (BW), body mass index (BMI), PRA, PAC, fasting plasma glucose (FPG), HbA1c, hematocrit (Ht), serum creatinine (s-Cr), estimated glomerular filtration rate (eGFR), and serum potassium (s-K) were collected as data. The examination of these parameters was performed using the standard methods. The plasma samples for PRA and PAC evaluation were collected into EDTA tubes between 0900 and 1000 h in the spine position for 30 min. PRA and PAC were measured using a radioimmunoassay [22]. Blood was collected in chilled EDTA tubes, and PRA and PAC measured by commercial radioimmunoassay (SRL, Tokyo, Japan). The values of inter- and intra-coefficient of variation in the measurement of PRA is lower than 10%, and in the measurement of PAC is 8.3 and 3.2% at 103 pg/mL. SBP, DBP, HR, and BW were measured in the examination rooms. SGLT2 inhibitors were orally administered to the study patients after breakfast once daily. We did not determine the specific types of SGLT2 inhibitors. We started the standard dosage: (empagliflozin 10 mg/day), 11 (ipragliflozin 50 mg/day), 10 (canagliflozin 100 mg/day), 9 (tofogliflozin 20 mg/day), 5 (dapagliflozin 5 mg/day), 4 and 1 (luseogliflozin 2.5 mg).

### Statistical analysis

Data are expressed as mean ± SD and were analyzed using commercially available statistical software (SPSS version 22.0 for Windows, IBM, Chicago, IL). *P* values < 0.05 were considered to indicate statistical significance. Variables determined before and after the administration of SGLT2 inhibitors were compared using a pairwise *t*-test. We calculated that sample size of 35 was necessary to provide up to 80% power to detect a difference in mean ARR level between two groups, assuming a mean difference of 40 and standard deviation of 100 according to previous study with significance of 0.05 and power of 80%.

The associations between each parameters and % changes in ARR were examined via Pearson correlation coefficient analysis. For variables with significant correlations, a multiple linear regression analysis was performed to identify independent factors, adjusting for sex, age, and BMI..

Patients were divided into two groups based on changes in ARR before and after SGLT2 inhibitor treatment. In the first group (elevated group), ARR was elevated after the administration of SGLT2 inhibitors, whereas in the second group (decreased group), ARR decreased after the administration of SGLT2 inhibitors. R Statistical Package (version 3.5.0) was used to perform random forest classifier [23]. A random forest classifier was trained with 80% of the data and tested on the remaining 20% using the “randomforest” package of R [24]. The parameters of random forest were age, sex, BMI, SBP, DBP, HR, FPG, HbA1c, Ht, s-Cr, eGFR, s-K. Random forest had an out-of-bag error rate of 57.5%; the classification error was 39.1%.

Listwise deletion was used to construct the sample for multiple linear regression analysis and random forest classifier, because all types of missing were missing completely at random.

## Results

### Baseline characteristics

Forty-eight patients met the inclusion criteria. In total, 40 patients remained in the analysis after the exclusion of two patients with PA and six patients taking MRA. The clinical characteristics (mean age, 58.3 ± 12.7 years, female rate, 50.0%) are summarized in Table 1. The number of daily anti-hypertensive medications was 1.2 ± 0.9, and the number of daily anti-hypoglycemic medications was 2.0 ± 1.2. The medicines taken by subjects with diabetes and hypertension are shown in Supplemental Table S1. Five patients did not receive anti-diabetic agents, except for SGLT2 inhibitors. Ten patients did not receive anti-hypertensive agents and fifteen patients did not receive angiotensin II receptor blockers (ARB), angiotensin converting enzyme inhibitors (ACEI), MRAs, diuretics, or beta-blockers. The number of patients with ARB/ACEI, diuretic, beta-blocker were 21, 4, and 1. One patient received a diagnosed of resistant hypertension, which is defined as BP > 140/90 mmHg in the general population or 130/80 mmHg in patients with diabetes or chronic kidney disease taking three or more anti-hypertensive agents or maintenance of normal BP with four or more anti-hypertensive medications [25].

**Table 1:** Evaluation before and after sodium–glucose cotransporter-2 inhibitor administration

	before	after	*p*-value
Body weight, kg	77.3 ± 18	76.0 ± 18	< 0.001
Body mass index, kg/m^2^	29.4 ± 5.5	28.9 ± 5.3	< 0.001
Systolic blood pressure, mmHg	130 ± 13	127 ± 13	N.S.
Diastolic blood pressure, mmHg	80 ± 10	77 ± 9	< 0.05
Heart rate, bpm	81 ± 12	76 ± 11	< 0.01
Hematocrit, mg/dL	42.4 ± 3.7	43.9 ± 3.6	N.S.
Serum creatinine, mg/dL	0.73 ± 0.18	0.76 ± 0.19	< 0.05
Estimated glomerular filtration rate, mL/min/1.73m^2^	81.3 ± 21.3	78.9 ± 21.1	N.S.
Serum potassium, mEq/L	4.1 ± 0.3	4.2 ± 0.3	N.S.
Fasting plasma glucose, mg/dL	157 ± 52	133 ± 29	< 0.01
HbA1c, %	8.1 ± 1.3	7.5 ± 1.0	< 0.001
Plasma renin activity, ng/mL/h	2.8 ± 3.0	3.5 ± 3.7	N.S.
Plasma aldosterone concentration, pg/mL	144 ± 127	155 ± 100	N.S.
Aldosterone-to-renin ratio	90.9 ± 51.6	81.4 ± 62.9	N.S.

Abbreviations: *BW* body weight, *BMI* body mass index, *SBP* systolic blood pressure, *DBP* diastolic blood pressure, *HR* heart rate, *Ht* hematocrit, *s-Cr* serum creatinine, *eGFR* estimate glomerular filtration rate, *s-K* serum potassium level, *FPG* fasting plasma glucose, *HbA1c* hemoglobin A1c, *ARR* aldosterone-to-renin ratio, *PRA* plasma renin activity, *PAC* plasma aldosterone concentration, *ARR* aldosterone to renin ratio, *N.S.* not significant

Data are n (%) or mean ± SD

### Changes in PAC, PRA, and ARR after the administration of SGLT2 inhibitors

The Change in PRA, PAC, and ARR after treatment with SGLT2 inhibitors are summarized in Table 1. After administering SGLT2 inhibitors, no significant changes were observed in the levels of PRA (2.8 ± 3.0 ng/mL/h vs. 3.5 ± 3.7 ng/mL/h), PAC (144 ± 127 pg/mL vs. 155 ± 100 pg/mL), and ARR (90.9 ± 51.6 vs. 81.4 ± 62.9). The Changes in biochemical and hemodynamic parameters following treatment with SGLT2 inhibitors are summarized in Table 1. There were 3 patients with ARR > 200 before or after SGLT2 inhibitor administration. They were excluded from PA by the captopril challenge test.

**Fig. 1:**
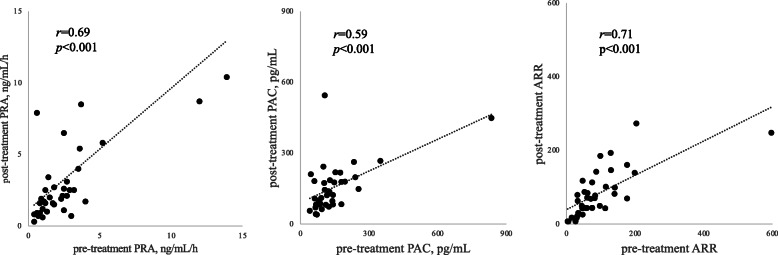
Correlation among PRA, PAC and ARR before and after SGLT2 inhibitor administration. Abbreviations: ARR, aldosterone-to-renin ratio; PRA, plasma renin activity; PAC, plasma aldosterone concentration; ARR, aldosterone to renin ratio

**Fig. 2:**
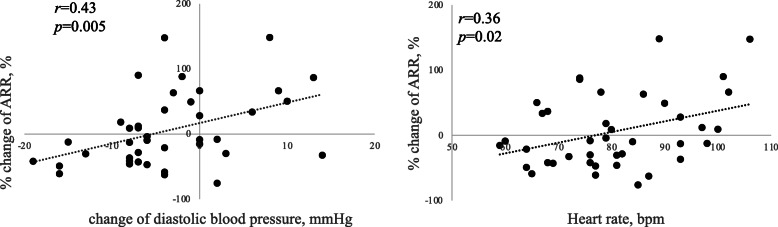
Relationship between percentage change in ARR and change in DBP or heart rate. Abbreviations: ARR, aldosterone-to-renin ratio; PRA, plasma renin activity; PAC, plasma aldosterone concentration; ARR, aldosterone to renin ratio

The correlation among PRA, PAC, and ARR before and after treatment is shown in Fig. 1. Figure 2 shows the Pearson’s correlation analysis, which revealed a significant relationship between % change of DBP or HR and change in ARR. The % changes of DBP (β = 0.422, *p* = 0.003) and HR (β = 0.502, *p* = 0.001) were significant predictors of change in ARR after adjusting for sex, age, and BMI at enrollment with multiple regression analysis; however, no significant relationship was found between the change in ARR and other markers.

### Comparison of parameters between elevated and decreased groups

Table 2 shows the clinical background at baseline and change in each parameter change after SGLT2 inhibitor treatment in the ARR elevated group and ARR decreased group. There were significant differences in DBP and HR changes. There was no difference in number of anti-hypertensive user between two groups. We ranked feature importance using the random forest algorithm and presented the results as percentage changes in DBP, and HR (Supplemental Fig. S1).

**Table 2:** Comparison of parameters between elevated and decreased groups

	Elevated Group (*n* = 17)		Decreased Group (*n* = 23)	
	Baseline	Change	Baseline	Change
Female, n (%)	8 (47)		11 (48)	
Age	59.1 ± 13.6		57.2 ± 12.5	
BW, kg	73.0 ± 19.3	−1.5 ± 1.9	80.5 ± 17	−1.2 ± 2.6
BMI, kg/m^2^	27.6 ± 4.4	−0.6 ± 0.8	30.7 ± 6.0	−0.5 ± 1.1
SBP, mmHg	129 ± 13	2 ± 15	130 ± 14	−6 ± 12
DBP, mmHg	79 ± 11	0 ± 7 *	81 ± 10	−6 ± 7
HR, bpm	85 ± 13 *	−6 ± 8	77 ± 11	−3 ± 8
Ht, mg/dL	41.3 ± 3.8	1.7 ± 2.1	43.8 ± 3.5	1.4 ± 1.8
s-Cr, mg/dL	0.74 ± 0.16	0.02 ± 0.06	0.72 ± 0.19	0.04 ± 0.08
eGFR, mL/min/1.73m^2^	78.4 ± 18.5	− 1.5 ± 10.6	83.4 ± 23.2	−3.6 ± 11.4
s-K, mEq/L	4.1 ± 0.3	0.1 ± 0.2	4.2 ± 0.3	0 ± 0.2
FPG, mg/dL	154 ± 42	−17 ± 37	159 ± 60	− 28 ± 51
HbA1c, %	8.2 ± 1.5	−0.8 ± 0.8	8.1 ± 1.2	−0.6 ± 0.8
PRA, ng/mL/h	3.3 ± 3.4	−0.2 ± 1.7	2.4 ± 2.7	1.4 ± 3.2
PAC, pg/mL	116 ± 56	30 ± 61	165 ± 161	−5 ± 134
ARR	67 ± 51	34 (8 to 60) ‡	109 ± 118	− 17 (− 48 to − 11)
Use of Ca-blocker, n (%)	8 (47)		11 (48)	
Use of Alpha-blocker, n (%)	1 (6)		2 (9)	
Use of beta-blocker, n (%)	1 (6)		0 (0)	
Use of ARB/ACEI, n (%)	8 (47)		13 (57)	
Use of diuretics, n (%)	2 (12)		2 (9)	

See Table 1 for abbreviations. Data are n (%), mean ± SD, or median (25th to 75th percentile)

*, *p* < 0.05; ‡, *p* < 0.001 vs. Decreased group

### Comparison of change in ARR and the duration after the administration of SGLT2 inhibitors

We analyzed the relationship between the change of ARR and duration after the administration of SGLT2 inhibitors. There was no correlation between the change of ARR and the duration after the administration of SGLT2 inhibitors (Supplemental Fig. 2).

### Changes in PAC, PRA, and ARR after the administration of SGLT2 inhibitors in patients without ARBs/ACEIs, diuretics, and beta-blockers, and MRA

For exclusion of the effects of ARBs/ACEIs, diuretics, and beta-blockers, and MRA, we analyzed 15 patients without these medications. The Change in PRA, PAC, and ARR after treatment with SGLT2 inhibitors in these group are summarized in Supplemental Table S2. No significant changes were observed in the levels of PRA (1.7 ± 1.1 ng/mL/h vs. 1.7 ± 1.2 ng/mL/h), PAC (139 ± 74 pg/mL vs. 141 ± 63 pg/mL), and ARR (107 ± 53 vs. 109 ± 67), which was similar results with the analysis intended for all patients.

## Discussion

Two to six months after administering the SGLT2 inhibitors, no significant changes were found in the levels of ARR. The change in ARR had a positive correlation with HR pretreatment and change in DBP.

Several clinical studies have evaluated the effects of SGLT2 inhibitors on systemic and intrarenal RAAS [26]. It was found that SGLT2 inhibitors result in systemic PRA elevation through osmotic diuresis at least in the early phase of treatment [18, 27]. In the chronic phase with more than 12 weeks after treatment, dapagliflozin increases PRA and serum aldosterone [28]. Conversely, PRA did not change significantly after 6 months of treatment in other studies [29, 30]. No clinical data have shown that ARR significantly changes by treatment with an SGLT2 inhibitor. However, in the kidney of patients with diabetes, SGLT2 inhibitors locally decrease renal angiotensinogen (AGT) expression by reducing glucose levels in the early proximal tubes [31], leading to increase in glucose load in the distal proximal tubule, which could increase AGT production [32]. Tuba et al. summarized the current available data indicating that treatment with SGLT2 inhibitors transiently activates systemic RAAS, but not intrarenal RAAS, in patients with type 2 diabetes [33]. To our knowledge, our study is the first to report the effects of SGLT2 inhibitors on ARR, one of the important systemic RAAS parameters in patients with diabetic patients with hypertension, which is clinically worthy to diagnose PA during screening.

Renal glucose reabsorption is increased in patients with diabetes [34]. In fact, renal glucose re-uptake is increased in the proximal tubular cells of these patients as measured by methyl-α-d-[U-^14^C]-glucopyranoside in renal proximal epithelial cells. In addition, the mRNA and protein expression of SGLT2 and GLUT2 are high in these patients [35]. SGLT2 inhibitors increase the urinary excretion of glucose by inhibiting glucose reabsorption in the proximal tubules of the kidney, thereby improving hyperglycemia [36]. With the use of these inhibitors, weight loss and hypotensive effects are expected, in addition to improvement in blood glucose level [33, 37].

Treatment with 10 mg dapagliflozin per day decreased body plasma volume to approximately 5.4% at 1 week after administration; however, this reduction was attenuated after 12 weeks [19]. Thiazide diuretics are a type of anti-hypertensive agents that often stimulate the renin–angiotensin system by decreasing extracellular fluid volume in patients with EHT [28]. Thiazide diuretics elevate both PRA and PAC, ultimately decreasing ARR, which may lead to false-negative results in subjects with hypertension [15]. This mechanism involves the inhibition of sodium ion reabsorption from the distal tubules of the kidney by blocking thiazide-sensitive Na^+^-Cl^−^ symporter and decreasing extracellular fluid volume [15]. Schork et al. reported the long-term effects of empagliflozin and dapagliflozin on fluid volume. By using bioimpedance spectroscopy, they found that there was no difference in the effects of empagliflozin and dapagliflozin when compared with those of hydrochlorothiazide treatment [38]. Although the action point and target channel in the kidney differs between SGLT2 inhibitors and thiazide diuretics, SGLT2 inhibitors can reduce extracellular fluid volume to the same extent as thiazide diuretics. In our study, changes in DBP and HR had a significant positive correlation with the changes in the ARR. Blood pressure is described by cardiac output and peripheral artery resistance. DBP is considered to be a marker of body fluid volume. An increase in the volume of body fluids results in an increase in cardiac output and participates in the elevation of blood pressure. Therefore, a positive correlation was found between the change in the ARR and DBP and random forest algorithm showed high importance of the change in DBP. This is because of the diuretic effect of SGLT2 inhibitors.

Several studies have indicated that SGLT2 inhibitors decrease blood pressure without causing an elevation in HR. Matsubayashi et al. reported that SGLT2 inhibitors decrease HR and this change in HR correlates with the baseline HR levels in patients with type 2 diabetes [39]. There are many reports on the effects of SGLT2 inhibitors on sympathetic nerve activity. Matthews et al. reported that the administration of dapagliflozin reduced thyrosine hydroxylase and noradrenaline, which is the marker of high sympathetic nerve activity in the kidney and heart in C57BL6/J mice [40]. This report indicated that the decrease in HR was because of the suppression of sympathetic nerve activity. Stimulating the sympathetic nerves can stimulate renin release by activating beta-1 stimulation in the renal juxtaglomerular apparatus [41]. In contrast, the suppression of sympathetic nerves activity decreases both renin and aldosterone release, ultimately increasing the ARR, a finding that is clinically similar to that found with beta- blocker treatment [13]. The sympathetic nerve activity of patients with a high HR pretreatment with SGLT2 inhibitors is stimulated and the degree of this activity is decreased greatly by these agents. Therefore, a positive correlation was found between the change in the ARR and HR and Random forest algorithm showed high importance of HR pretreatment. Thus, SGLT2 inhibitors may have false-positive effects on the screening of PA in the patients with a high HR.

Our study had the following limitations:

1) We adopted a retrospective design and therefore, data for the control groups were not obtained, the sample size was also small.

2) The impact of various classes of antihypertensive medications on ARR might have interfered to some degree with the interpretation of our results. However, previous studies proved that ARR is an effective screening tool even in patients receiving anti-hypertensive treatment [42, 43].

3) ARRs were evaluated 2 to 6 months after the administration of SGLT2 inhibitor in our study. The change of ARR had correlation with SGLT2 inhibitor induced diuretic effect and sympathoinhibition effect. The sympathoinhibition by SGLT2 inhibitor was reported from early phase of SGLT2 inhibitor and continue to delayed phase of SGLT2 inhibitors [44]. Conversely, the diuretic effect of SGLT2 inhibitors were greater in early phase compared with that in delayed phase [18, 20]. The greater diuretic effect of SGLT2 inhibitors may have larger effect on decrease in ARR compared with that in delayed phase. However, we have no data about the change of ARR within 2 months after administration of SGLT2 inhibitors. The evaluation of ARR in the early phase of SGLT2 inhibitor is future task.

4) The target patients in our study had poor diabetic control. Nevertheless, improving blood glucose level, diabetic state, and anti-diabetic agents might have some effects on the RAAS. In fact, pioglitazone, a thiazolidinedione, was reported to suppress the production of aldosterone [45, 46]. Liraglutide, a GLP-1 agonist, may also suppress the RASS [47]; nonetheless, the effects of anti-diabetic agents on ARR have not been reported. Most patients with PA are also prescribed medications for diabetes, rather than for hypertension alone. Therefore, further studies are needed to evaluate the effect of anti-diabetic agents on ARR in PA screening.

## Conclusions

We revealed that ARR did not significantly change after treatment with SGLT2 inhibitors. SGLT2 inhibitors neither significantly reduced ARR nor increased the risk of false-negative results during PA screening.

## Supplementary Information

**Additional file 1: Supplemental Table S1**. Medication for subjects with hypertension or diabetes. Abbreviations: ARB, angiotensin II receptor blocker; ACEI, angiotensin converting enzyme inhibitor. Data are n (%).

**Additional file 2: Supplemental Table S2**. Effect of sodium–glucose cotransporter-2 inhibitor administration in patients without ARBs/ACEIs, diuretics, and beta-blockers, and MRA. See Table 1 for abbreviations. Data are n (%), mean ± SD.

**Additional file 3: Supplemental Fig. S1**. Feature importance ranking to categorize elevated ARR group and decreased ARR group. The explanatory variables were BW, body weight; SBP, systolic blood pressure; DBP, diastolic blood pressure; HR, heart rate; FPG, fasting plasma glucose; HbA1c, hemoglobin A1c; Ht, hematocrit; s-Cr, serum creatinine; eGFR, estimate glomerular filtration rate; s-K, serum potassium level; age; sex.

**Additional file 4: Supplemental Fig. S2**. Correlation between the change in ARR and the duration after the administration of SGLT2 inhibitor. Abbreviations: ARR, aldosterone-to-renin ratio.

## Data Availability

The data are available from the corresponding author upon reasonable request.

## Abbreviations

ARR: Plasma aldosterone-to-renin ratio
PA: Primary aldosteronism
SGLT2: Sodium glucose cotransporter 2
EHT: Essential hypertension
IGT: Insulin glucose tolerance
HbA1c: Hemoglobin A1c
RAAS: Renin angiotensin aldosterone system
PRA: Plasma renin activity
PAC: Plasma aldosterone concentration
MRA: Mineralocorticoid receptor antagonist
SBP: Systemic blood pressure
DBP: Diastolic blood pressure
HR: Heart rate
BW: Body weight
BMI: Body mass index
FPG: Fasting plasma glucose
Ht: Hematocrit
s-Cr: Serum creatinine
eGFR: Estimated glomerular filtration rate
s-K: Serum potassium
AGT: Angiotensinogen
