# Supplementary material for: Effect of sodium–glucose cotransporter-2 inhibitors on aldosterone-to-renin ratio in diabetic patients with hypertension: a retrospective observational study
Source: BMC Endocr Disord. 2020 Nov 30;20:177. doi: 10.1186/s12902-020-00656-8 (PMC7706199; doi:10.1186/s12902-020-00656-8)
Supplement: Supplementary file 1 — Additional file 1: Supplemental Table S1. Medication for subjects with hypertension or diabetes. Abbreviations: ARB, angiotensin II receptor blocker; ACEI, angiotensin converting enzyme inhibitor. Data are n (%). [file 12902_2020_656_MOESM1_ESM.docx]

| **Supplemental Table S1.** |  |
| --- | --- |
| Use of anti-hypertensive agents, n (%) | 30 (75.0) |
| Ca-blocker, n (%) | 19 (47.5) |
| Alpha-blocker, n (%) | 3 (7.5) |
| Beta-blocker, n (%) | 1 (2.5) |
| ARB/ACEI, n (%) | 21 (52.5) |
| Diuretics, n (%) | 4 (10.0) |
| Use of insulin, n (%) | 9 (22.0) |
| Use of anti-diabetic agents, n (%) | 33(84.6) |
| SGLT2 inhibitors |  |
| Empagliflozin, n (%) | 11 (27.5) |
| Ipragliflozin, n (%) | 10 (25.0) |
| Canagliflozin, n (%) | 9 (22.5) |
| Dapagliflozin, n (%) | 5 (12.5) |
| Tofogliflozin, n (%) | 4 (10.0) |
| Luseogliflozin, n (%) | 1 (2.5) |
