# Supplementary material for: Effect of sodium–glucose cotransporter-2 inhibitors on aldosterone-to-renin ratio in diabetic patients with hypertension: a retrospective observational study
Source: BMC Endocr Disord. 2020 Nov 30;20:177. doi: 10.1186/s12902-020-00656-8 (PMC7706199; doi:10.1186/s12902-020-00656-8)
Supplement: Supplementary file 2 — Additional file 2: Supplemental Table S2. Effect of sodium–glucose cotransporter-2 inhibitor administration in patients without ARBs/ACEIs, diuretics, and beta-blockers, and MRA. See Table 1 for abbreviations. Data are n (%), mean ± SD. [file 12902_2020_656_MOESM2_ESM.docx]

| **Supplemental Table S2.** | | | |
| --- | --- | --- | --- |
|  | before | after | *P*-value |
| Body weight, kg | 71.9 ± 17 | 70.8 ± 17 | <0.001 |
| Body mass index, kg/m^2^ | 28.5 ± 6.6 | 28.1 ± 5.9 | <0.001 |
| Systolic blood pressure, mmHg | 127 ± 13 | 130 ± 12 | N.S. |
| Diastolic blood pressure, mmHg | 78 ± 12 | 77 ± 9 | <0.05 |
| Heart rate, bpm | 81 ± 12 | 77 ± 12 | <0.01 |
| Hematocrit, mg/dL | 43.6 ± 3.2 | 44.4 ± 3.8 | N.S. |
| Serum creatinine, mg/dL | 0.70 ± 0.18 | 0.72 ± 0.18 | <0.05 |
| Estimated glomerular filtration rate, mL/min/1.73m^2^ | 83.6 ± 21.1 | 84.1 ± 21.1 | N.S. |
| Serum potassium, mEq/L | 4.1 ± 0.2 | 4.1 ± 0.3 | N.S. |
| Fasting plasma glucose, mg/dL | 158 ± 61 | 131 ± 24 | <0.01 |
| HbA1c, % | 8.3 ± 1.6 | 7.9 ± 1.1 | <0.001 |
| Plasma renin activity, ng/mL/h | 1.7 ± 1.1 | 1.7 ± 1.2 | N.S. |
| Plasma aldosterone concentration, pg/mL | 139 ± 74 | 141 ± 63 | N.S. |
| Aldosterone-to-renin ratio | 107 ± 53 | 109 ± 67 | N.S. |
