# Supplementary figures and images for: Effect of sodium–glucose cotransporter-2 inhibitors on aldosterone-to-renin ratio in diabetic patients with hypertension: a retrospective observational study
Source: BMC Endocr Disord. 2020 Nov 30;20:177. doi: 10.1186/s12902-020-00656-8 (PMC7706199; doi:10.1186/s12902-020-00656-8)

Supplemental Figure S1.

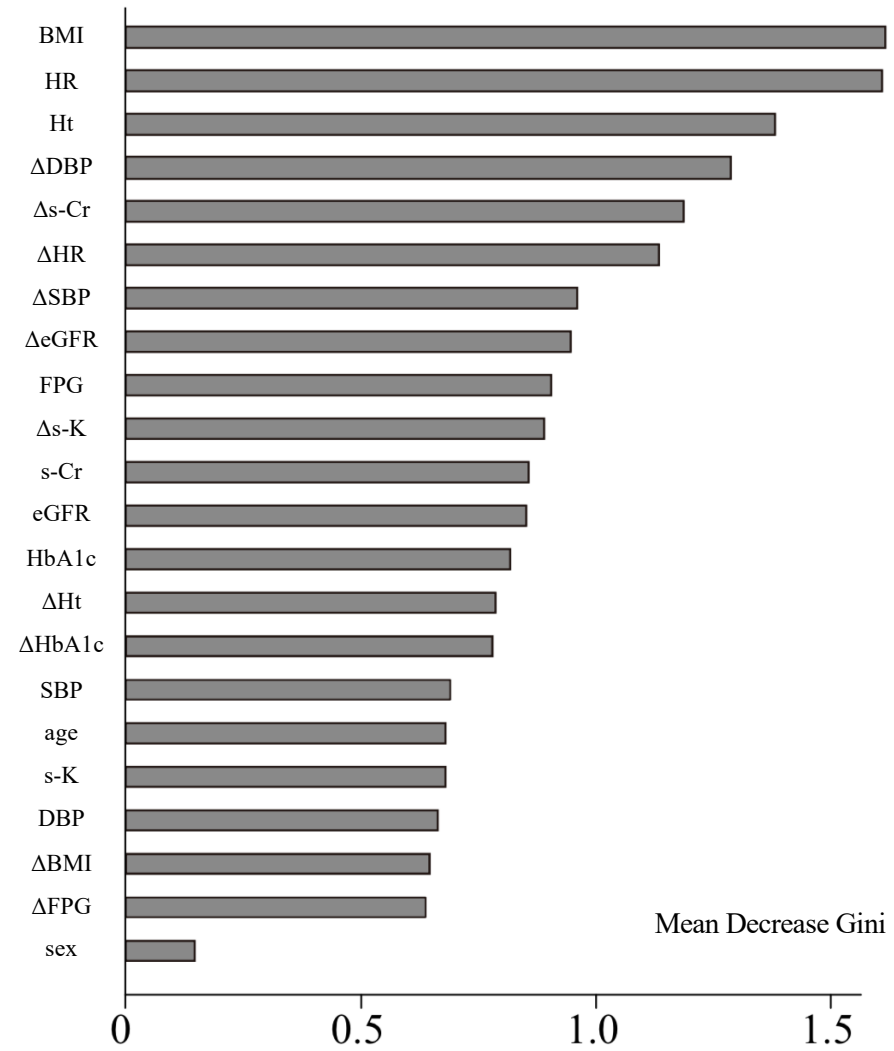

Supplement: Supplementary file 3 — Additional file 3: Supplemental Fig. S1. Feature importance ranking to categorize elevated ARR group and decreased ARR group. The explanatory variables were BW, body weight; SBP, systolic blood pressure; DBP, diastolic blood pressure; HR, heart rate; FPG, fasting plasma glucose; HbA1c, hemoglobin A1c; Ht, hematocrit; s-Cr, serum creatinine; eGFR, estimate glomerular filtration rate; s-K, serum potassium level; age; sex. [file 12902_2020_656_MOESM3_ESM.pdf]

**Supplemental Figure S2.**

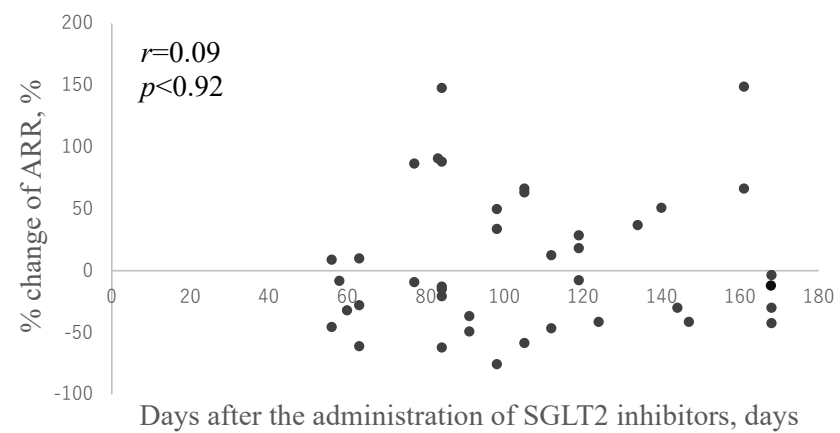

Supplement: Supplementary file 4 — Additional file 4: Supplemental Fig. S2. Correlation between the change in ARR and the duration after the administration of SGLT2 inhibitor. Abbreviations: ARR, aldosterone-to-renin ratio. [file 12902_2020_656_MOESM4_ESM.pdf]
